# Molecular Characterization of *Mycobacterium avium* subsp. *hominissuis* of Two Groups of Lymph Nodes, Being Intradermal Tuberculin or Interferon-Gamma Test Positive and Negative, Isolated from Swiss Cattle at Slaughter

**DOI:** 10.3389/fvets.2018.00032

**Published:** 2018-03-05

**Authors:** Simone Scherrer, Patricia Landolt, Natasha Carroli, Roger Stephan

**Affiliations:** ^1^Vetsuisse Faculty, Institute of Veterinary Bacteriology, University of Zurich, Zurich, Switzerland; ^2^Vetsuisse Faculty, Institute for Food Safety and Hygiene, University of Zurich, Zurich, Switzerland

**Keywords:** *Mycobacterium avium* subsp. *hominissuis*, variable number of tandem repeat, ISMav6, hsp65 code, ITS1 sequevar, cattle, lymph nodes

## Abstract

*Mycobacterium avium* subsp. *hominissuis* (MAH) is an important zoonotic pathogen with raising global health concerns. In humans, MAH is one of the most widespread non-tuberculous mycobacterial species responsible for lung disease. In animals, MAH is frequently isolated from pigs; however, it is also an opportunistic pathogen for other mammals including cattle. To elucidate the genetic diversity of MAH in cattle, a molecular characterization of isolates (*n* = 26) derived from lymph nodes was performed. Fourteen isolates originated from slaughtered cattle with visible altered lymph nodes at meat inspection, whereas 12 isolates were from lymph nodes without any gross pathological changes of healthy slaughtered cattle. Variable number of tandem repeat (VNTR) analysis was performed at 20 loci to examine genetic differences of isolates and to compare to previously reported VNTR data of human isolates from different countries. Genetic elements IS901, IS1245, IS1311, LSPA17, ITS1 sequevar, and hsp65 code were determined. Interestingly, two bovine MAH isolates harbored ISMav6 and hsp65 code 15, which so far has only been observed in human isolates. We supposed that VNTR data of Swiss samples would show clustering with European samples. Minimum spanning tree and unweighted pair group method using arithmetic averages analyses based on the VNTR data indicated a specific cluster of MAH isolates obtained from lymph nodes without any gross pathological changes of healthy slaughtered cattle. Comparing Swiss isolates with isolates from different other countries, no geographical clustering was observed; however, four Swiss isolates had an identical VNTR profile as human isolates from the Netherlands, the United States, and Japan. These findings indicate a possible public health issue.

## Introduction

Non-tuberculous mycobacteria (NTM) are ubiquitous in the environment, mainly in water and soil (1). Recently, more and more new NTM species were discovered, probably because of improved culturing techniques and the development of new molecular methods. Currently, more than 150 *Mycobacterium* species are known (1). *Mycobacterium avium* subsp. *hominissui*s (MAH), a representative of the *M. avium* complex (MAC), is an environmental bacterium often found in water, soil, dust, or straw, and its main hosts are humans and pigs. It is also an opportunistic pathogen for other mammals including cattle, from which it is one of the most frequently isolated NTM (2, 3). *M. avium* infection in humans is leading to severe symptoms, whereas pigs are often subclinically infected (4). An experimental infection of goats with MAH performed by Schinköthe et al. demonstrated two different courses of disease with highly heterogenic lesions, systemic spread in goats with severe clinical disease and the development of granulomas of all stages in the surviving goats (5). Recently in Asia, a Japanese black beef steer was discovered to have MAH granulomas in the systemic organs, indicating that MAH can cause systemic mycobacteriosis in cattle (6). Especially in developed countries, MAH is frequently linked to human mycobacteriosis (7–9). Turenne et al. showed that MAH has the highest level of genomic heterogeneity within MAC. They concluded that *M. avium* subsp. *paratuberculosis* (MAP), *M. avium* subsp. *avium* (MAA), and *M. avium* subsp. *silvaticum* (MAS) evolved independently from MAH (10). Zoonotic aspects of MAH have been investigated to explore exposure risk for humans. Recent population wide genetic studies (11–13) have shown that MAH isolates from Japanese patients had a low degree of similarity with the Japanese pig isolates, whereas the isolates from European patients and pigs showed a high genetic relatedness with the Japanese pig isolates. In contrast to the human cases, pig isolates were more homogenous. To compare MAH isolates across geographical regions and in different hosts, variable number of tandem repeat (VNTR) typing analysis based on the eight loci [mycobacterial interspersed repetitive unit (MIRU)–VNTR] was developed (14). Another VNTR panel of 15 loci [*M. avium* tandem repeats (MATRs)–VNTR] was introduced (15). Both MIRU- and MATR-VNTR panels are useful for global epidemiological studies. VNTR has a high level of reproducibility and has the advantage of allowing a numerical and reproductive digitalization of typing data and therefore enabling an optimal comparison of results between laboratories (16).

To investigate the molecular features of Swiss bovine MAH, a collective of MAC isolates was gathered from two previous studies. Since MAH seemed to be the predominant NTM species in Swiss cattle, the aim of this study is to map molecular characteristics of MAH isolated from lymph nodes of cattle at slaughter. VNTR data were compared to previously reported data from different countries to elucidate genetic relatedness of isolates from Swiss cattle with international human isolates and to assess geographical differences in the genetic diversity of MAH.

## Materials and Methods

### Ethics Statement

This study was carried out in accordance with the recommendations of Swiss federal regulations (TSV 916.401 and VSFK 817.190). Analysis of animal specimens was carried out within an official context of monitoring bovine tuberculosis and NTM infections, meaning that no animals were killed for the purposes of this research project and ethical approval was not necessary.

### Collection of Isolates

Due to some tuberculosis cases in the cattle population in Switzerland, altered lymph nodes from 534 cattle, tested suspicious or positive by either using intradermal tuberculin test alone or in combination with interferon-γ testing, were submitted between March 2013 and November 2014 for mycobacteriological analysis (group A). An average of three lymph nodes was processed from each animal. Lymph nodes were of retropharyngeal, mandibular, bronchial, or mediastinal origin. *Mycobacterium tuberculosis* complex (MTBC)-negative cultures resulting in 57 NTM isolates were obtained, and of those, 14 (24.6%) isolates were identified to be MAH. Furthermore, 108 lymph nodes without any gross pathological changes were collected from healthy slaughtered cattle in 2015 giving rise to 20 NTM cultures including 12 (60%) MAH isolates (group B). These 26 MAH isolates were included in this study.

### Mycobacterial Culture and DNA Extraction

Mycobacterial culture was performed as described previously (17). Genomic DNA was extracted harvesting mycobacteria from 1.5 ml MGIT cultures by centrifugation for 10 min at 13,000 × *g*. The sediment was suspended in 180 µl ATL buffer (Qiagen, Hilden, Germany), transferred onto a bead beating matrix in a 2-ml microtube (Omni International, Kennesaw, USA), heat inactivated, and subjected to mechanical cell lysis using a TissueLyser II (Qiagen) and enzymatic digestion with Proteinase K (Qiagen). Automated DNA preparation was performed on the QIAcube instrument using the QIAamp cador Pathogen Mini Kit protocol (Qiagen). DNA concentration was measured using a NanoDrop 2000c Spectrophotometer (Thermo Fisher Scientific, Reinach, Switzerland) and stored at −20°C until use. Standard biosecurity procedures have been carried out for handling of samples.

### Identification and Characterization of MAH

For identification of mycobacteria, 16S rRNA [16S rRNA forward primer: TTGGAGAGTTTGATCMTGGCTC (adapted from CLSI MM-18-A), sequencing primer 259: TTTCACGAACAACGCGACAA (18) and 16S rRNA reverse primer 1492R: TACGGYTACCTTGTTACGACTT (19)], *rpoB* (20), and *hsp65* (21) were sequenced. Sequence analysis of the 3′ fragment of the *hsp65* gene was performed by amplifying the near-complete *hsp65* gene using primers MAC*hsp65*F and MACh*sp65*R. To confirm the resulting *hsp65* code based on the single-nucleotide polymorphism, Sanger sequencing was performed in duplicate (22). All isolates including four reference strains (MAH ATCC 700898, MAP ATCC 19698, MAA ATCC 25291 and MAS ATCC 49884) were tested by PCR for the presence of the IS*901* element as described previously (22). Detection and sequencing of IS*Mav6* was performed by PCR using specific primer sets designed for MAH (8). Sequencing of the ITS1 region was performed as described previously (23, 24). Results were compared to ITS1 sequences in GenBank including Mav-A (GenBank accession number EF521901.1), Mav-B (GenBank accession number L07856), and Mav-F (GenBank accession number AF315838) sequences (7, 25). Large sequence polymorphisms (LSPs) are molecular markers of genetic diversity in both MTBC (26) and *M. avium* subspecies (27). LSP^A^17 was tested on all MAH isolates trying to differentiate presumable MAA being positive for IS*901* from MAH, since the absence of LSP^A^17 is supposed to be characteristic for MAA strains, although not perfectly specific (28). IS*1245* and IS*1311* were detected according to the study by Johansen et al. (9).

The nucleotide sequences of 16S rRNA, *rpoB* and *hsp65* genes obtained were compared with available sequences by BLAST analysis using the NCBI database.^1^ Alignment of sequences was performed using CLC Main Workbench 7.6.1 (CLC Bio, Qiagen, Germany).

### VNTR Analysis

Variable number of tandem repeat typing was performed on 26 MAH isolates by amplification of 15 MATR (MATR-1, 2, 3, 4, 5, 6, 7, 8, 9, 11, 12, 13, 14, 15, and 16) (15) and 8 MIRU (MIRU-292, X3, 25, 47, 3, 7, 10, and 32) (14) loci as described earlier. Three loci overlapped between MIRU-VNTR and MATR-VNTR: MIRU-292 and MATR-2, MIRU-X3 and MATR-3, and MIRU-10 and MATR-9. Each reaction mixture contained 1× HotStart *Taq* Master Mix Kit, 1× Q-Solution (Qiagen) (only for loci MATR-5, 8, 9, 11, and 12 and MIRU-47), 0.5 µM of each primer pair and 1 ng purified mycobacterial DNA in a final volume of 10 µl. PCR was performed with an initial 15-min activation step at 95°C followed by 40 cycles of 95°C for 30 s, 60°C for 30 s, 72°C for 30 s, and a final extension step of 72°C for 10 min. 10 µl of each PCR amplification products were analyzed using a capillary electrophoresis device (QIAxcel, Qiagen), applying OH1700 AM10sec method with a QX DNA high-resolution cartridge, QX 15 bp–3 kb alignment marker and QX 100 bp–2.5 kb size marker. Peak size assignment and allele code exportation were performed with the QIAxcel ScreenGel Software version 1.3.0 (Qiagen). As a positive control, four reference strains (MAH ATCC 700898, MAP ATCC 19698, MAA ATCC 25291, and MAS ATCC 49884) were analyzed for each PCR run. For the MATR loci, the number of tandem repeats was determined from the size of amplicons. Repeat numbers (alleles) of MIRU-VNTR loci were assigned according to a previously described allele-calling table and arranged to profiles, called INMVs (INRA Nouzilly MIRU-VNTR), described by Thibault et al. (14). New profiles detected were registered in the MAC-INMV database.^2^ The allelic diversity index (h) of the different loci was calculated as described by Mazars et al. (29). The Hunter-Gaston Discriminatory Index (HGDI), representing a numerical index for the discriminatory power of a genotyping method, was determined according to the formula described by Hunter and Gaston (30).

### Phylogenetic Analysis

Genetic strain lineages were predicted using online tools available from MIRU-VNTR*plus* website^3^ (31). A dendrogram based on 20 VNTR loci was generated using the unweighted pair group method using arithmetic averages (UPGMA) algorithm.

Variable number of tandem repeat data from a previous report (11) was retrieved for the geographical comparison of genetic diversity of MAH. A minimum spanning tree (MST) was generated based on 14 loci VTNR genotype data that combined our own data of the 26 Swiss isolates with previously published data using online tools available from MIRU-VNTR*plus* website. The MST classification into three main subgroups (Cluster A, B, and C) was adopted (11).

## Results

### Molecular Characterization of MAH

PCR for IS*901* yielded positive results for the two reference strains MAA ATCC 25291 and MAS ATCC 49884. The IS*901* PCR also produced an amplicon for MAH isolates ZH38 and ZH43 belonging to group B. Sequencing of the obtained PCR product from these isolates revealed a 100% sequence identity with the recently described IS*Mav6* from Japan (GenBank accession number AB447556.1). PCR for IS*901* yielded negative results for all remaining MAH isolates including reference strain MAH ATCC 700898 and MAP ATCC 19698. PCR amplification of the ITS1 region resulted in a single amplicon of 480 bp for all MAC isolates. Sequence analysis revealed three *M. avium* sequevars, Mav-A, Mav-B, and Mav-F, whereas 21 isolates belonged to Mav-B (80.8%), 3 isolates to Mav-F (11.5%), and 2 isolates to Mav-A (7.7%). The major *hsp65* sequevars of MAH isolates from Swiss cattle was code 1 (23.1%) and code 2 (65.4%), less frequently code 15 (7.7%) and a newly identified code N8 (3.8%) (Table 1). LSP analysis showed 12 isolates (46.2%) without LSP^A^17 and 14 isolates (53.8%) with LSP^A^17. Insertion element analysis revealed IS*1245* positive for all except one isolate and IS*1311* positive for all except two isolates (Table 2).

**Table 1:** Single-nucleotide polymorphisms among 26 Swiss *Mycobacterium avium* subsp. *hominissuis* isolates of this study in comparison with *Mycobacterium avium* 104.

hsp65 code	Nucleotide position of *hsp65* in strain*M. avium* 104																				Distribution
	240	276	324	612	633	645	861	927	1092	1128	1136	1218	1269	1272	1296	1350	1435	1468	1488	1536	
1	C	G	C	C	C	C	G	C	G	C	C	A	G	C	C	G	A	G	G	A	6 (23.1%)
2	·	·	T	·	·	·	·	·	·	·	·	G	·	·	·	·	·	·	·	G	17 (65.4%)
15	·	·	·	·	·	·	·	T	·	·	·	·	·	·	·	·	·	·	·	A	2 (7.7%)
N8^a^	·	·	T	·	·	·	·	·	·	·	·	·	·	·	·	·	·	·	·	G	1 (3.8%)

*Matching residues are marked as dots*.

*^a^New code type found in this study was designated as code N8*.

**Table 2:** Molecular characteristics of 26 Swiss *Mycobacterium avium* subsp. *hominissuis* (MAH) isolates evaluated in this study.

Species	Isolate	Cluster^a^	INMV profiles^b^	IS*901*	ITS1 sequevar	*hsp65* code	LSP^A^17	IS*1245*	IS*1311*
MAH	ZH38	Cluster A East Asian Type	INMV 207	+ (ISMav6)	Mav-F	15	−	−	−
MAH	ZH43		INMV 207	+(ISMav6)	Mav-F	15	−	+	+
MAH	ZH74		INMV 210	−	Mav-F	2	−	+	−
MAH	NTM11		INMV 211	−	Mav-B	2	+	+	+
MAH	NTM51		INMV 104	−	Mav-B	1	+	+	+

MAH	ZH22	Cluster B	INMV 205	−	Mav-B	N8	+	+	+
**MAH**	**ZH34**		**INMV 206**	−	**Mav-B**	**2**	**+**	**+**	**+**
**MAH**	**NTM21**		**INMV 206**	−	**Mav-B**	**2**	**+**	**+**	**+**
**MAH**	**NTM32**		**INMV 206**	−	**Mav-A**	**2**	**+**	**+**	**+**
MAH	ZH48		INMV 208	−	Mav-B	2	+	+	+
MAH	ZH49		INMV 208	−	Mav-B	2	+	+	+
MAH	NTM26		INMV 208	−	Mav-B	2	+	+	+
MAH	ZH64		INMV 209	−	Mav-B	2	+	+	+
MAH	NTM18		INMV 212	−	Mav-B	2	+	+	+
MAH	ZH9		INMV 213	−	Mav-B	2	+	+	+
MAH	ZH46		INMV 167	−	Mav-A	2	−	+	+
MAH	ZH100		INMV 213	−	Mav-B	2	−	+	+
MAH	NTM39		INMV 206	−	Mav-B	1	+	+	+
MAH	ZH24		INMV 206	−	Mav-B	1	+	+	+

MAH	NTM19	Cluster C	INMV 123	−	Mav-B	2	−	+	+
MAH	NTM20		INMV 123	−	Mav-B	2	−	+	+
MAH	NTM49		INMV 123	−	Mav-B	2	−	+	+
MAH	NTM52		INMV 123	−	Mav-B	2	−	+	+
**MAH**	**NTM34**		**INMV 92**	−	**Mav-B**	**1**	−	**+**	**+**
**MAH**	**NTM46**		**INMV 92**	−	**Mav-B**	**1**	−	**+**	**+**
MAH	NTM6		INMV 51	−	Mav-B	1	−	+	+

*Bold indicates common VNTR code with international isolates*.

*^a^Classification into clusters according to Ichikawa et al. (11)*.

*^b^Denominated according the INRA Nouzilly MIRU-VNTR patterns as INMV profile (14). Nine new profiles (INMV 205–INMV 213) were identified*.

By assigning all molecular characteristics (ITS1 sequevar, *hsp*65 code, LSP^A^17, IS*1245*, IS*1311*) to geographical clusters distinctive features could be observed. Cluster C had most homogenous properties with ITS1 sequevar Mav-B: the absence of LSP^A^17 and the presence of IS*1245* and IS*1311* and *hsp65* code of 1 or 2. Cluster A was heterogeneous; ITS1 sequevar was Mav-F and Mav-B, *hsp65* code was 15, 2, and 1, and LSP^A^17, IS*1245*, and IS*1311* were present or absent. In Cluster B, the distribution of molecular characteristics was heterogeneous as well; IS*1245* and IS*1311* were present, ITS1 sequevar was Mav-A or Mav-B, *hsp65* code was either N8, 2, or 1, and LSP^A^17 was mostly present except for two isolates (Table 2).

### VNTR Analysis

MIRU-VNTR analysis resulted in 14 profiles, called INMVs. Nine profiles (INMV 205–INMV 213) were newly identified. Diversity of combined genotypes was eight INMV profiles for both groups A and B. Two genotypes were shared (INMV 206 and INMV 208) by both groups. Eleven genotypes were only detected in one or two isolates. The three most frequently detected genotypes in this study were INMV 206, INMV 123, and INMV 208 (Table S1 in Supplementary Material).

**Figure 1:**
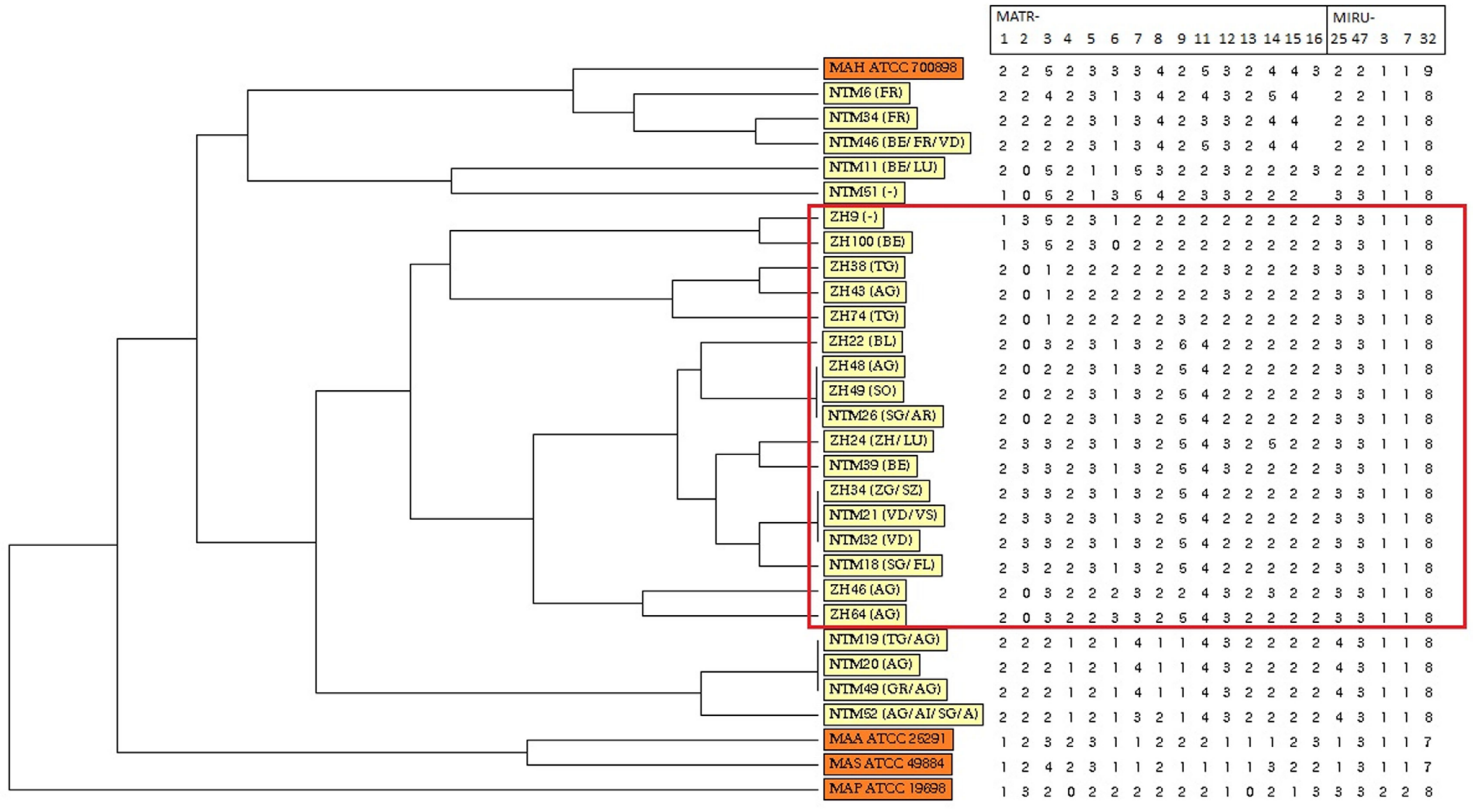
Dendrogram and allele profiles constructed from variable number of tandem repeat typing results of 26 Swiss *Mycobacterium avium* subsp. *hominissuis* (MAH) isolates including four reference strains. The dendrogram was generated with the unweighted pair group method using arithmetic average algorithm using tools available from the MIRU-VNTR*plus* database. Reference strains MAA ATCC 25291, MAS ATCC 49884, MAH ATCC 700898, and MAP ATCC 19698 are colored in orange, whereas all MAH isolates of this study are colored in yellow. The red box highlights a cluster of isolates from lymph nodes without any gross pathological changes of healthy, slaughtered cattle (group B).

Variable number of tandem repeat profiles including both 15 MATR loci and 5 MIRU loci from 26 MAH isolates (Table S1 in Supplementary Material) were used to calculate the allelic diversity (Table 3). In terms of discriminatory hierarchy loci MATR-3, MATR-9, MATR-2, MATR-7, and MATR-5 displayed the highest allelic diversity. In contrast, MATR-13, MIRU-3, MIRU-7, and MIRU-32 were monomorphic (Table 3). Three triplicates of isolates with identical VNTR profiles (ZH34, NTM21, NTM32; ZH48, ZH49, NTM26 and NTM19, NTM20, NTM49) were observed, resulting in 20 distinct allelic VNTR profiles (Figure 1). The HGDI was 0.972 revealing a good discriminatory power of the VNTR analysis.

**Table 3:** MATR/MIRU allelic distribution and diversity among 26 Swiss *Mycobacterium avium* subsp. *hominissuis* isolates evaluated in this study.

Locus	Number of isolates with a specific number of tandem repeats (allele)										Allelic diversity (h)^a^
	0	1	2	2.5	3	4	5	6	7	8	
MATR-1	0	3	23	0	0	0	0	0	0	0	0.17
**MATR-2**	11	0	7	0	8	0	0	0	0	0	**0.64**
**MATR-3**	0	3	10	0	8	1	4	0	0	0	**0.71**
MATR-4	0	4	22	0	0	0	0	0	0	0	0.23
**MATR-5**	0	2	9	0	15	0	0	0	0	0	**0.52**
MATR-6	1	19	4	0	2	0	0	0	0	0	0.41
**MATR-7**	0	0	5	0	16	3	2	0	0	0	**0.55**
MATR-8	0	3	18	0	1	4	0	0	0	0	0.46
**MATR-9**	0	4	10	0	1	0	10	1	0	0	**0.66**
MATR-11	0	0	6	0	2	17	1	0	0	0	0.49
MATR-12	0	0	11	0	15	0	0	0	0	0	0.47
MATR-13	0	0	26	0	0	0	0	0	0	0	0
MATR-14	0	0	12	0	1	2	2	0	0	0	0.31
MATR-15	0	0	23	0	0	3	0	0	0	0	0.17
MATR-16	0	0	20	4	2	0	0	0	0	0	0.35
MIRU-3	0	26	0	0	0	0	0	0	0	0	0
MIRU-7	0	26	0	0	0	0	0	0	0	0	0
MIRU-25	0	0	4	0	18	4	0	0	0	0	0.45
MIRU-47	0	0	4	0	22	0	0	0	0	0	0.23
MIRU-32	0	0	0	0	0	0	0	0	0	26	0

*Bold indicates an allelic diversity ≥0.5*.

*^a^VNTR allelic distribution was calculated as described by Mazars et al. (29)*.

### Phylogenetic Analysis

**Figure 2:**
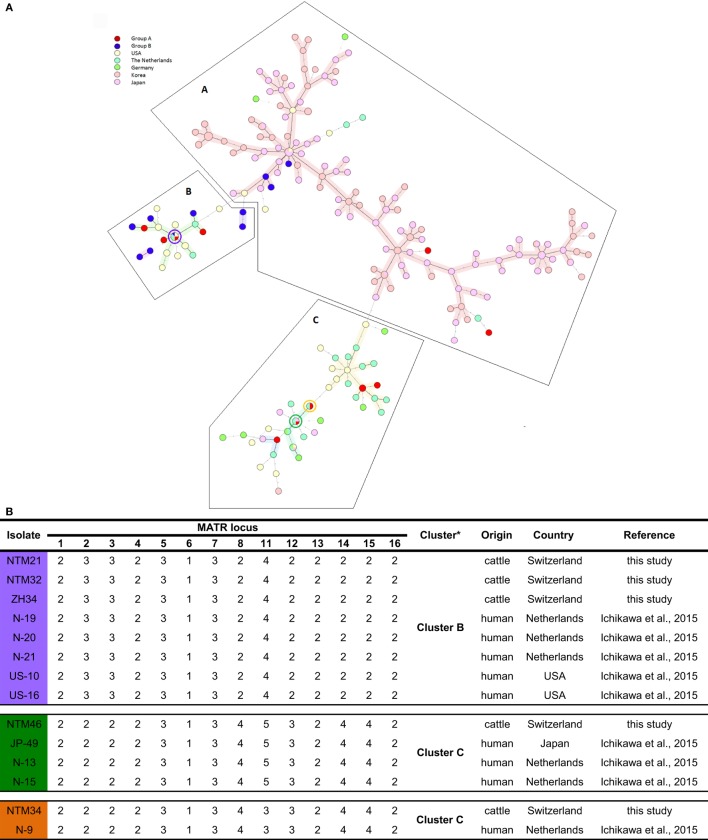
**(A)** Representation of Swiss *Mycobacterium avium* subsp. *hominissuis* (MAH) isolates in an international context using a minimum spanning tree based on the 14-MATR-VNTR genotyping of MAH isolates (26 isolates from Switzerland, 94 isolates from Japan, 98 isolates from Korea, 32 isolates from the United States, 27 isolates from The Netherlands, and 10 isolates from Germany). Red (group A) and blue (group B) represent Swiss isolates. Violet, green, and orange circles represent Swiss isolates with identical profiles to Dutch, American, and Japanese isolates. **(B)** VNTR profiles of Swiss isolates with common international VNTR profiles. *Classification in clusters based on MATR-VNTR proposed by Ichikawa et al. (11).

Unweighted pair group method using arithmetic average analysis displayed a cluster of isolates deriving from lymph nodes without any gross pathological changes (Figure 1). MST analysis of MAH isolates from six different countries revealed a scattered distribution of Swiss isolates (Figure 2A).

Among the population of isolates of group B, a predominant grouping in Cluster A (25%; 3/12) and Cluster B (75%; 9/12) was found. In Cluster C, none of those isolates were found. In contrast, for isolates of group A, a distribution in all three clusters was observed: Cluster A consisted of 14.3% (2/14), Cluster B of 35.7% (5/14), and Cluster C of 50% (7/14) of MAH isolates. In total, 19.2% (5/26) Swiss isolates were grouped into Cluster A, which represents the East Asian Type mainly, and 53.9% (14/26) isolates in Cluster B together with the United States and The Netherlands and 26.9% (7/26) in Cluster C, which is the most heterogeneous cluster harboring isolates from Asia, Europe, and the United States. As anticipated, the two IS*Mav6*-positive isolates (ZH38 and ZH43) were found in Cluster A together with the isolates of Japan and Korea.

Four Swiss isolates showed an identical VNTR profile compared to isolates of different European and East Asian countries. NTM21 and NTM32 were identical to isolates N-19, N-20, and N-21 from the Netherlands and to isolates US-10 and US-16 from the United States; NTM34 was identical to isolate N-9 from the Netherlands, and NTM46 was identical to isolate JP-49 from Japan and to isolates N-13 and N-15 from the Netherlands (Figure 2B).

## Discussion

This study showed a good discriminatory power of PCR-based VNTR analysis and the usefulness to compare resulting VNTR genetic profiles using global sample sets. The UPGMA and MST analysis presented a genetically heterogeneous set of Swiss isolates and a widespread geographical distribution compared to other countries.

To investigate the genetic relatedness of MAH isolates in greater detail, UPGMA analysis was preformed based on 20 loci VNTR profile of the isolates. A cluster of MAH isolates deriving from lymph nodes of group B was revealed, indicating a possible relationship between VNTR profile and virulence. Comparing each VNTR locus of isolates of group A and B, it could be observed that allelic distribution of five loci (MATR-4, MATR-8, MATR-15, MIRU-25, and MIRU-47) was monomorphic (Table S1 in Supplementary Material) for isolates from group B in comparison to isolates from group A. However, one limitation of this study is that group A and group B were determined by gross analysis of the lymph nodes. Therefore, no validation of group designations has been performed.

A partial geographical correlation could be observed between the main clusters and the origin of MAH isolates. In one case, animals collected on the same farm (NTM19, NTM20, and NTM49) revealed one distinct VNTR profile. Of cattle showing an identical VNTR profile (NTM21, NTM32, and ZH34), only two cattle (NTM21 and NTM32) were from the same canton, but not from the same farm. Cattle harboring ZH48, ZH49, and NTM26 with an identical VNTR profile were in non-neighboring cantons. On the other hand, there were cattle (NTM6 and NTM34) originating from the same canton but showing different distinct profiles. Furthermore, one case of a geographical cluster with three animals of the same farm (NTM19, NTM20, and NTM49) could be observed. The three isolates showed an identical VNTR profile, indicating a common infection source. The affected cattle were slaughtered at the age of 5 and 6 months and 1 year, respectively, indicating an infection at young age. A fourth cow (NTM52) from the same farm showed a variation of VNTR profile in MATR-7 and MATR-8. This cow had a trading history within Switzerland and Austria. Presumably, the infection source could be within the farm from an animal with a mixed infection of two MAH subtypes or it could arise from a closely related MAH genotype present in the nearby region. Heterogeneity within a sample possibly containing different subtypes was not studied since only one colony of the original culture was subcultured. While studying the trading histories of involved animals, no other clusters reflecting a direct contact between cattle could be discovered. An absence of correlation between allelic profiles and geographical sample source of isolates could be explained in indirect transmission of MAH through common environmental sources, other animals, or humans. Several epidemiological studies of MAH in Europe found no correlation between genetic profiles and geographical origins, as well (32–36).

IS*Mav6*, a homologous insertion sequence to the original bird-type IS*901* with 95% sequence identity (8), was found in two MAH isolates comprising *hsp65* sequevar code 15. This combination of IS*Mav6* together with *hsp65* sequevar code 15 has been described for Japanese clinical isolates often recovered from sputum (8, 37) and more recently from Korean clinical isolates, where a high prevalence of IS*Mav6* was observed (38). Adachi et al. compared IS*Mav6* in human and swine isolates detecting IS*Mav6* in human samples only (39). In a human patient from Germany, one MAH isolate with *hsp65* code 15 harboring IS*Mav6* was reported (40). In Belgium, three human isolates containing IS*Mav6* were found, and one of those isolates was IS*1245* positive and two were IS*1245* negative. However, *hsp65* sequevar of those Belgian isolates was code 1 and 2, respectively (41). As IS*901* is believed to have an influence on the pathogenicity of MAA (42, 43), IS*Mav6* may also be related to the pathogenicity of MAH (8). Interestingly, in this study, IS*Mav6* was detected for the first time from animal origin.

Remarkably, in Cluster B and C, four Swiss isolates showed identical VNTR profiles compared to isolates from the Netherlands, the United States, and Japan. Identical VNTR genetic profiles could be an indication for epidemiological linkage. Or ultimately, homologous genotypes may have evolved in parallel. Among the 5 Swiss MAH isolates belonging to Cluster A, two isolates with similar 14 loci VNTR profiles to East Asian isolates were IS*Mav6* positive, indicating that MAH isolates could have been introduced to Switzerland form East Asia. Nevertheless, isolates with identical VNTR profiles do not necessarily perfectly reflect phylogenetic relationship because there could be differences in other genetic elements, indicating only distantly related isolates. This could be observed in isolates ZH34, NTM21, and NTM32 sharing the same VNTR copy numbers but different ITS1 sequevar, namely Mav-B for NTM21 and NTM32 and Mav-A for ZH34. Consequently, VNTR could give some insight in the epidemiology of strains evolving worldwide and its connections; however, data should be critically analyzed. To globally analyze VNTR data, it is of advantage to use an extended set of VNTR loci rather than reducing VNTR data to a country specific set of loci with high allelic diversity and to include other molecular characteristics as ITS1 and *hsp65* sequevar. We would propose combining MATR and MIRU loci into a single panel of 20 loci VNTR, serving as global standard for international comparison of genotyping data, thereby minimizing false assignment of epidemiological linkage. With such a creation of an international VNTR data base, it could be possible to better understand the global pattern of MAH genetics and therefore achieve better control in MAH infections.

## Conclusion

We could show a clustering in the distribution of VNTR profiles of isolates from lymph nodes without any gross pathological changes of healthy slaughtered cattle in comparison to isolates from slaughtered cattle with visible altered lymph nodes indicating a possible link to virulence. Further studies elucidating subtractive differences of the genome of MAH isolates of healthy cattle versus isolates of animals with altered lymph nodes would give rise to some insight into differences in virulence.

The geographical comparison of genetic diversity of MAH indicated no distinct distribution of Swiss isolates related to international clusters. Remarkably, some Swiss isolates showed an identical VNTR code in 14 loci as in human isolates causing pulmonary diseases from the Netherlands, the United States, and Japan. Moreover, the detection of IS*Mav6*, which was observed for the first time in animals and otherwise, is frequently distributed in human isolates in the East Asian part of the world, underlines a great genomic variability of Swiss MAH isolates from cattle.

## Author Contributions

SS and RS designed and coordinated the study. PL, NC, and SS performed the experiments. SS and PL interpreted the data. PL sketched the figures. SS drafted the manuscript. RS reviewed and edited the manuscript. All authors read and approved the final manuscript.

## Conflict of Interest Statement

The authors declare that the research was conducted in the absence of any commercial or financial relationships that could be construed as a potential conflict of interest.

## Footnotes

^1^https://www.ncbi.nlm.nih.gov/.

^2^http://mac-inmv.tours.inra.fr/.

^3^www.miru-vntrplus.org.
